# Indirect sinus lift without bone graft material: Systematic review and meta-analysis

**DOI:** 10.4317/jced.51716

**Published:** 2015-04-01

**Authors:** Sara Pérez-Martínez, Luis Martorell-Calatayud, David Peñarrocha-Oltra, Berta García-Mira, Miguel Peñarrocha-Diago

**Affiliations:** 1 Master in Oral Surgery and Implant Dentistry, Stomatology Department, Faculty of Medicine and Dentistry, University of Valencia, Spain; 2 Collaborating Professor of the Master in Oral Surgery and Implant Dentistry, Stomatology Department, Faculty of Medicine and Dentistry, University of Valencia, Spain; 3 Associate Professor of Oral Surgery, Stomatology Department, Faculty of Medicine and Dentistry, University of Valencia, Spain; 4 Chairman of Oral Surgery, Stomatology Department, Faculty of Medicine and Dentistry, University of Valencia, Spain

## Abstract

A systematic literature review and a meta-analysis of indirect sinus lift without the use of bone graft material was performed. A PubMed search was made from January 2005 to January 2012 with keywords: “sinus lift”, “osteotome”, “graft” and “maxillary sinus elevation”. The inclusion criteria were: maxillary sinus lift technique with osteotomes with a minimum follow-up period of 5 months after surgery without bone graft material. 11 articles were included. The mean gain in residual crestal bone height after maxillary sinus lift without bone graft material was 3,43 mm ± 0,09 (2,5 mm – 4,4 mm). The survival rate ranged from 94% to 100%. Placement of implants with sinus lift without bone graft material, is a valid surgical technique to gain residual crestal height and placed implants in an atrophic posterior maxillary with a crestal height from 5 to 9 mm.

** Key words:**Sinus lift, osteotome, graft, maxillary sinus elevation.

## Introduction

An important requirement for the correct placement of the implant is the presence of an adequate quantity and quality of residual bone (1). In 1994 Summers introduced the sinus lift technique with the use of osteotomes combined with graft material around the implant. This technique is a well-validated surgical option for situations with limited residual bone height; ≥ to 5-6 mm (2-4). The survival rate of implants placed simultaneously with indirect sinus lift with bone graft material ranges between 93.5% and 100% (5-6).

The bone graft material allows to keep the volume of the sinus membrane after performing indirect elevation (7). The choice of material has been controversial for years to authors. Recently, several studies have reported favorable results when performing indirect sinus lifts without the use of any bone graft (7-11). The authors agree that graft material is not necessary to promote osseointegration and maintain optimal bone volume around the implant, while the absence of graft reduces the risk of infections (12).

The aim of this study was to systematically review the literature regarding the treatment outcome of indirect sinus lift without graft material according to bone height gained after placement of dental implants.

## Material and Methods

A systematic search was conducted in the PubMed database for articles published between January 2005 and January 2012 on indirect sinus lift without the use of bone graft material. The following keywords individually or in combination were used: “sinus augmentation” OR “sinus elevation” OR “sinus lift” AND “indirect” OR “transcrestal”. Titles and abstracts were reviewed to identify relevant studies.

-Inclusion criteria:

• Use of indirect sinus elevation in the atrophic maxilla with osteotomes and without the use of bone graft material

• Minimum follow-up of 5 months after surgery

• Studies reporting at least survival rate of implants placed after the sinus lift procedure

• Full text in English or Spanish

-Exclusion criteria:

• Systematic reviews

• Clinical cases

• Studies on direct sinus lift or comparing between direct and indirect elevation

The initial search yielded 289 articles. 193 were excluded after checking the title. 85 additional studies were excluded after checking the abstract: 8 for being literature reviews, 14 for being series of clinical cases, 12 reported the performance of indirect sinus elevation with techniques different from the osteotome technique, 7 for not providing all the required variables, 12 for being studies on direct sinus lift, 19 for involving the use of bone graft material and 13 for being in a language different from English or Spanish. Full texts were reviewed for a total of 12 articles.

The following variables were registered: number of patients, age at the time of the intervention, number of implants placed, follow-up period, residual bone height before the sinus lift procedure, height gained to the sinus, implant survival rate and pe-ri-implant marginal bone loss ( Table 1) (8,9,13-20).

**Table 1:**
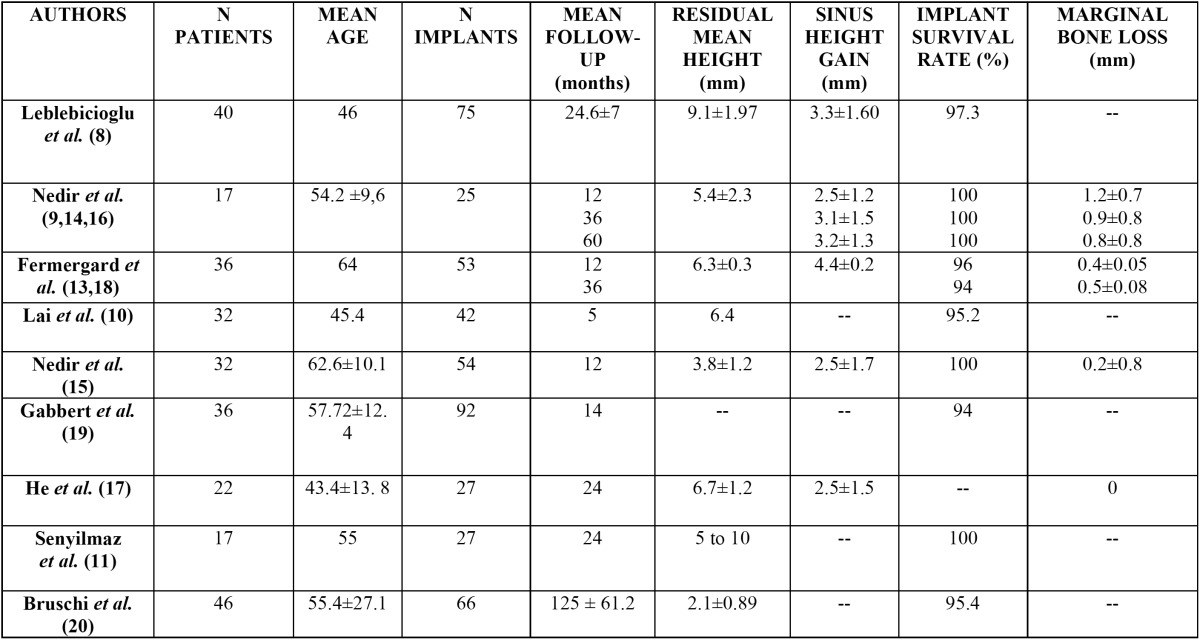
Studies included in the review.

Articles reported by the same research group on the same sample of patients at different follow-up times were grouped; data with the longest follow-up were used for the statistical analysis. Three articles by Nedir *et al.* (9,13,14) and 2 by Fermergard *et al.* (15,16) were grouped.

-Statistical analysis

A meta-analysis was performed to estimate the overall gain to the maxillary sinus, but it could not be performed for implant survival due to the lack of homogeneity in the follow-up time between studies. The estimate was based on the average weighted by the inverse of the variance, using a confidence interval of 95%. The significance level used in the analysis was 5% (α = 0.05).

## Results

The 8 included studies grouped a total of 461 implants in 278 patients.

-Sinus gain

Eight of the reviewed articles (8,9,13-18) involving 5 different patient samples reported sinus height gained after indirect elevation ( Table 2). The lowest sinus height gained was reported by the three studies on the same patient sample (9,17,18) with 2.5 mm; the greatest height gain was 4.4 mm and it was reported by Fermergard *et al.* in 2 articles on the same sample (15,16). Nedir *et al.* (9,13,14) measured the sinus height gained 1, 3 and 5 years after performing indirect sinus lift without graft, and reported increasing values of 2.5 ± 1.2 mm, 3.1 ± 1.5 mm and 3.2 ± 1.3 mm respectively. Fermergard *et al.* (15,16) conducted a similar study and reported the same sinus height gain (4.4 ± 0.2 mm) after 1 and 5 years. Leblebicioglu *et al.* (8) obtained 3.3 ± 1.60 mm of sinus height gain after a mean follow-up of 24.6 ± 7 months.

**Table 2:**
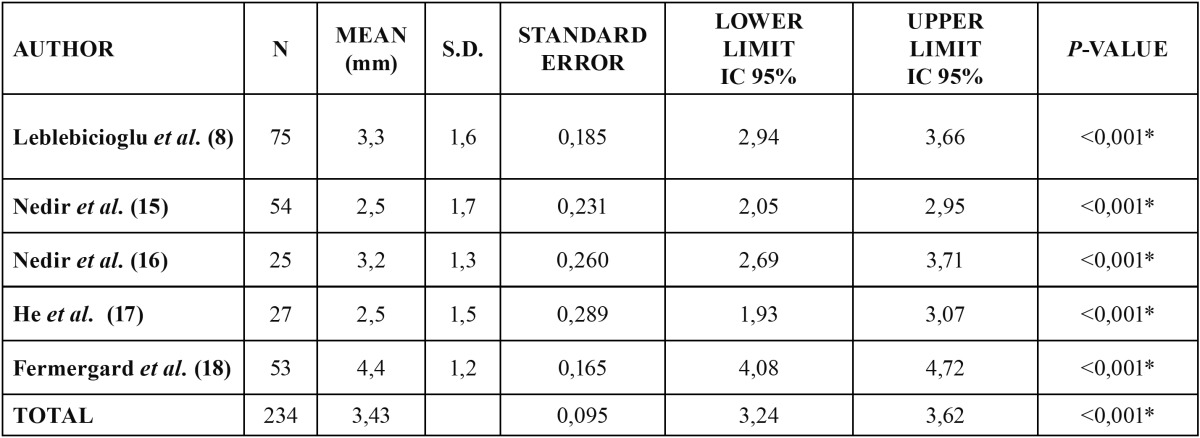
Statistical analysis of the gain to the sinus.

 Table 2 describes the bone gain outcomes of each study and the combined outcomes for the 5 studies.

In all studies, the gain to the maxillary sinus was significantly non-zero (*p* <0.001). The mean overall gain calculated for all studies was 3.43 mm, with a standard error of 0.095 and a confidence interval of 95% (3.24 to 3.62).

-Survival rate of implants 

The survival rate of the implants was studied in all the 12 articles and ranged from 94% to 100%. The lowest survival rates were reported by two studies: Gabbert *et al.* (19) reported six failures out of 92 implants placed (implant survival rate of 93.5%), all caused by a lack of osseointegration during the first 6 months. Fermergard *et al.* (15) obtained a survival rate of 94% after a follow up of three years.

Some of the articles reviewed, reported the survival rate of the implants and the mean residual bone height, which allowed to study implant survival depending on the residual bone height (8-11,13-16,20). Nedir et al. (9,13,14) recorded a survival rate of 100% after 1, 3 and 5 years, with a residual average height of 5.4 mm. Senyilmaz *et al.* (11) obtained a survival rate of 100% at 2 years for residual bone heights ranging between 5 and 10 mm. Fermergard *et al.* (15) reported two failures (survival rate 96%) with a residual bone of 6.3 ± 0.3 mm. After a period of three years, the same authors (16) recorded another failed implant, obtaining a survival rate of 94%. Bruschi *et al.* (20) studied the survival rate with the lowest residual bone height (2.11 ± 0.89 mm). Three failed implants were reported yielding a survival rate of 95.4% after a mean follow-up of 10.43 ± 5.01 years. Lai *et al.* (10) and Leblebicioglu *et al.* (8) reported survival rates of 95.2% and 97.3% with bony residual ridges of 6.4 ± 1.97 mm and 9.1 mm respectively.

## Conclusions

The limited evidence available suggests that indirect sinus lift without the use of bone graft material could be a valid technique to treat with implants atrophic posterior maxillae with residual heights between 5 and 9 mm. The reviewed studies reported a mean bone height gain of 3.43 ± 0.09 mm and implant survival rates ranging between 93.5% and 100%. However, more studies, with bigger samples, providing controlled groups treated with bone graft material and involving evaluation with cone beam computed tomographic scans performed at different timepoints are necessary.
